# Unsaturation-Driven Modulation of Antioxidant and Acetylcholinesterase Inhibitory Activities of Cardanol Derivatives

**DOI:** 10.3390/bioengineering12121316

**Published:** 2025-12-01

**Authors:** Roberta Bussons Rodrigues Valério, Halisson de Souza, Vitor Martins, Katherine Silva, Jane Eire de Manezes, Anderson Chaves, Leonardo F. Serafim, Antônio Vieira-Neto, José Cleiton S. dos Santos, Selene de Morais

**Affiliations:** 1 Postgraduate Program in Natural Sciences, State University of Ceará—UECE, Itaperi Campus, Fortaleza 60714-903, CE, Brazil; rbussons@hotmail.com (R.B.R.V.);; 2 Laboratory of Natural Products Chemistry, State University of Ceará—UECE, Itaperi Campus, Fortaleza 60714-903, CE, Brazil; 3 Advanced Materials Chemistry Group (GQMAT), Department of Analytical and Physical Chemistry, Federal University of Ceará—UFC, Pici Campus, CP 12100, Fortaleza 60451-970, CE, Brazil; 4 Department of Chemistry, Georgia State University, Atlanta, GA 30302, USA; 5 Center for Experimental Biology, University of Fortaleza, Av. Washington Soares, 1321, Fortaleza 60811-905, CE, Brazil; aevneto@gmail.com; 6 Institute of Engineering and Sustainable Development (IEDS), University of International Integration of Afro-Brazilian Lusophony—UNILAB, Campus das Auroras, Rua José Franco de Oliveira, s/n, Redenção 62790-970, CE, Brazil

**Keywords:** cardanol, spectroscopy, antioxidant, acetylcholinesterase, molecular dynamics, docking

## Abstract

**Background:** Cardanol, a bioactive alkylphenol obtained from technical cashew nutshell liquid (CNSL), consists of mono-, di-, and tri unsaturated side chain derivatives. These compounds are of increasing interest for their diverse industrial applications. **Methods:** In this study, three major cardanol components—3-[(8E)-8-pentadecen-1-yl] phenol (C1), 3-[(8E,11E)-8,11-pentadecadien-1-yl] phenol (C2), and 3-[(8E,11E,14E)-8,11,14-pentadecatrien-1-yl] phenol (C3)—were isolated from CNSL via chromatographic separation. Their structures were confirmed by FTIR and detailed ^1^H and ^13^C NMR spectroscopy, with complete carbon and proton assignments reported. **Results:** Among the three, C3 exhibited the highest antioxidant potential, with a DPPH IC_50_ value of 0.179 ± 0.005 mg/mL, approximately five times more potent than C1 (IC_50_ = 1.000 ± 0.200 mg/mL). C3 also showed the highest lethality against *Artemia salina* (LC_50_ = 4.118 ± 0.328 mg/mL) and the most pronounced inhibition of acetylcholinesterase (AChE), with a 0.8 cm inhibition zone. Computational studies using molecular docking and molecular dynamics simulations further supported the interaction of these compounds with both the catalytic active site (CAS) and the peripheral anionic site (PAS) of AChE. **Conclusions:** These results expand our understanding of the chemical composition and potential applications of CNSL. The identified unsaturated alkylphenols, particularly the triene compound, demonstrate promise as sustainable candidates for the development of new materials and pharmaceuticals that combat oxidative stress and neurodegenerative diseases.

## 1. Introduction

Cashew nutshell liquid (CNSL) is a viscous, dark brown byproduct obtained from cashew nuts (*Anacardium occidentale*) processing. It constitutes approximately 25% of the total cashew weight (0–35% to the weight of the shell) and is a significant component of the shell [1,2]. CNSL has gained considerable interest for its diverse industrial applications, including the production of resins, brake linings, paints, primers, foundry chemicals, lacquers, cement, and biofuels [3,4,5].

Additionally, it is a valuable source of bioactive compounds with pharmaceutical and cosmetic applications. Compounds derived from CNSL exhibit a wide range of biological activities, including antimicrobial, fungicidal, insecticidal, termiticidal, antioxidant, anticancer, and enzymatic inhibition properties [6,7,8,9,10,11].

**Scheme 1:**
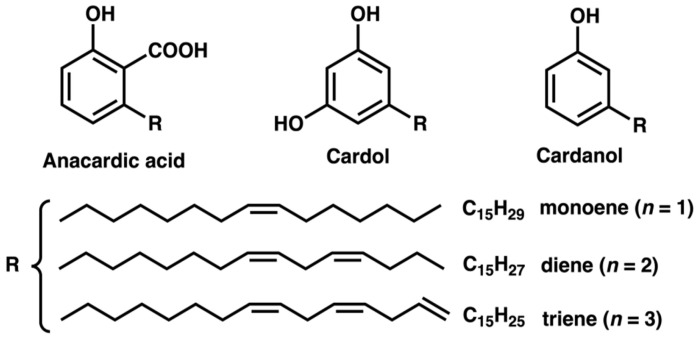
Chemical structures of the main constituents of Brazilian natural and technical cashew nutshell liquid.

Chemically, CNSL is a phenolic mixture composed primarily of anacardic acid, cardol, and cardanol, as depicted in Scheme 1. However, its composition varies significantly depending on the extraction method. Cold extraction yields “natural CNSL,” comprising 46–65% anacardic acid, 15–31% cardol, 10–22% cardanol, and trace amounts of methyl-cardol. In contrast, hot extraction produces “technical CNSL” [12,13,14].

The anacardic acids are thermally unstable and are readily decarboxylated during the hot extraction process. Therefore, their concentration in the technical CNSL will vary depending on the time and the temperature to which the cashew nut was submitted. In general, the main composition of the technical CNSL is: 1–2% of anacardic acid, 4–19% of cardol, 60–95% of cardanol [12,15,16].

The phenol group is responsible for the radical scavenging activity observed in many phytochemicals, often surpassing the antioxidant efficacy of vitamins E and C. Moreover, phenolic compounds such as cardanol exhibit enhanced antioxidant activity due to their long alkyl side chains, which stabilize oxidized molecules and inhibit further radical formation. Additionally, their lipophilic nature enables them to cross the blood–brain barrier, allowing antioxidant activity in the central nervous system [17,18,19].

Corroborating recent studies that highlighted the dual antioxidant and acetylcholinesterase inhibitory potential of phenolic-rich natural extracts and synthetic derivatives. The phytochemical profile of *Phytolacca acinosa* berries was analyzed, and abundant phenolic compounds with potent radical scavenging and AChE-inhibiting effects were found, surpassing those of the leaves [20]. Similarly, benzyloxychalcone hybrids, which are potential multifunctional AChE inhibitors, showed greater binding compatibility in silico than galantamine, a clinically used drug [21]. Such evidence reinforces the promise of phenolic structures as models for neuroprotective drug discovery, which aligns with the bioactivity trends observed for cardanol derivatives in this study.

Alzheimer’s disease (AD) is a multifactorial neurodegenerative disorder. Acetylcholinesterase (AChE) plays a key role in the regulation of the cholinergic system and particularly in the formation of amyloid plaques; therefore, the inhibition of AChE has become one of the most promising strategies for the treatment of AD. Inhibitors of AChE may be classified into two types: those binding to catalytic active site (CAS) and those binding peripheral catalytic site (PAS) Galantamine is a CAS binder [22] while donepezil spans CAS and PAS [23]. These have attracted attention, given that it has been described how AChE through PAS interacts with Aβ peptides, stimulating their aggregation [24].

However, despite the abundance of research on cardanol, the structure-activity association between the degree of unsaturation and the dual antioxidant/inhibitory potential of AChE remains underexplored. In this study, the cardanol constituents of technical CNSL were analyzed using chromatography, infrared spectroscopy, and NMR spectroscopy. Three unsaturated cardanol derivatives were isolated from the hexane extract of CNSL. Their antioxidant properties (DPPH radical inhibition), and acetylcholinesterase (AChE) inhibition were evaluated. These findings expand our understanding of CNSL’s chemical composition and potential applications. The identified unsaturated alkyl phenols show promise as valuable raw materials for the development of novel materials and pharmaceutical products.

## 2. Materials and Methods

### 2.1. Solvents and Reagents

Technical CNSL was supplied by Companhia Industrial de Óleos do Nordeste (CIONE-Fortaleza, CE, Brazil). Methanol, hexane, ethyl acetate, dimethyl sulfoxide (DMSO), hydrochloric acid, and 1,1-diphenyl-2-picrylhydrazyl (DPPH) were obtained from Dynamics Química Contemporânea Ltda. (Sao Paulo, SP, Brazil). 5,5′-Dithiobis-(2-nitrobenzoic acid) (DTNB), acetylthiocholine iodide (ACTI), silver nitrate (AgNO_3_), and acetic acid were purchased from Sigma-Aldrich (St. Louis, MO, USA). Acetonitrile (HPLC grade) was obtained from J.T. Baker (Mexico City, Mexico). Silica gel (0.063–0.200 mm, 70–230 mesh) was purchased from Macherey-Nagel (Düren, Germany).

### 2.2. Extraction and Separation of Cardanol Compounds from Technical Cashew Nutshell Liquid

The extraction of cardanol was previously carried out by the study group, following an established method for obtaining the compound [10]. The cardanol mixture (30 g) was fractionated by silver ion complexometric chromatography on a column using 300 g of silica gel impregnated with AgNO_3_. The silica gel was prepared by dissolving AgNO_3_ in 125 mL of water, mixing with the silica, and drying at 75 °C for three days in a light-protected vessel before use. The column was initially eluted with pure hexane, followed by increasing the polarity of the mobile phase with ethyl acetate and methanol in varying proportions. The fractions collected were analyzed via thin-layer chromatography (TLC) and recombined based on their retention factors. To improve chromatographic resolution and allow for the efficient separation of saturated and unsaturated constituents, silver nitrate was used to impregnate the silica gel, since Ag^+^ ions can form temporary and reversible π complexes, mainly olefins (alkenes), with the carbon-carbon double bonds in the unsaturated side chains of cardanol. This interaction strengthens the retention of unsaturated compounds in the stationary phase [25].

### 2.3. High-Performance Liquid Chromatography

HPLC analysis was performed using a Shimadzu SPD-10VP system (Shimadzu Corporation, Tokyo, Japan) with a UV-VIS detector. The chromatographic conditions were as follows: Hypersil GOLD column (25 cm), run time of 25 min, detection wavelength of 280 nm, flow rate of 1.80 mL/min, and a mobile phase consisting of 80% acetonitrile and 20% of a 1% (*v*/*v*) aqueous acetic acid solution.

### 2.4. Spectroscopic Analysis

The cardanol constituents: 3-[(8E)-8-pentadecen-1-yl] phenol (C1), 3-[(8E,11E)-8,11-pentadecadien-1-yl] phenol (C2), and 3-[(8E,11E,14E)-8,11,14-pentadecatrien-1-yl] phenol (C3) were analyzed via ^1^H and ^13^C nuclear magnetic resonance (NMR) spectroscopy in deuterated chloroform at 70 °C using a BRUKER AVANCE DRX-500 MHz spectrometer (500 MHz for ^1^H, 125 MHz for ^13^C) (Bruker, Billerica, MA, USA). Fourier-transform infrared (FTIR) spectra were recorded using a Shimadzu FTIR-8300 spectrometer. All samples (cardanol and its main constituents) were analyzed in the solid state using the KBr pellet technique. The samples were carefully macerated in an agate mortar and pestle to ensure homogeneity, then mixed with spectroscopic-grade KBr in a 1:10 (sample:KBr, *w*/*w*) ratio. The mixtures were pressed under vacuum into transparent pellets using a hydraulic press before measurement. Spectra were collected in the 4000–400 cm^−1^ range under ambient conditions.

### 2.5. Determination of DPPH Radical Scavenging Activity

Antioxidant activity was assessed using the DPPH radical scavenging assay [26]. Different concentrations of each compound were mixed with a methanol solution of DPPH (6.5 × 10^−5^ M) in test tubes. After 60 min, absorbance was measured at 515 nm using a UV-Vis spectrophotometer. Experiments were performed in triplicate, and inhibition percentage (*IP%*) was calculated using the equation:

(1) $$IP\%=\left[\left(I_{DPPH}-I_{S}\right)/I_{DPPH}\right]\times 100$$

In Equation (1), *I_DPPH_* represents the absorbance of the DPPH solution and *I_S_* represents the absorbance of the sample-containing solution. The results were compared with that of quercetin, the standard antioxidant [8].

### 2.6. Assessment of Anticholinesterase Activity

The anticholinesterase activity of cardanol compounds was evaluated using TLC coupled with Ellman’s reagent. The TLC plates were sprayed with a solution of DTNB and ATCI, followed by 3 U/mL of acetylcholinesterase (AChE). Enzyme inhibition was observed as white spots on a yellow background. The diameter of these spots was measured 10 min after appearance. Physostigmine was used as a positive control.

### 2.7. Brine Shrimp Lethality Assay

The lethality assay against *Artemia salina* Leach was conducted following a previously reported methodology with adaptations [27]. The *A. salina* eggs hatched in water with salinity of 12 ppm and after 48 h, the larvae were collected for bioassays. Dilutions of samples and a blank test were prepared in methanol or in a mixture of DMSO (1% *v*/*v* DMSO) in sea water. Triplicate sample solutions were prepared to be tested at concentrations of 1000, 100, 10, and 1 ppm. Ten nauplii were added to each jar, and the counting of surviving larvae was made 24 h later.

### 2.8. Statistical Analysis

The results from the experiments are reported in the following format: means ± standard deviation (SD). One-way analysis of variance (ANOVA) was used to determine statistical differences, followed by Tukey’s multiple comparison test in GraphPad Prism (version 9.5.1; GraphPad Software, San Diego, CA, USA) at 5% probability. The LC_50_ values were calculated using Microsoft Excel (Microsoft Office 365, version 2408; Microsoft Corp., Redmond, WA, USA).

The results from the experiments are reported in the following format: means ± standard deviation (SD). One-way analysis of variance (ANOVA) was used to determine statistical differences, followed by Tukey’s multiple comparison test in GraphPad Prism (version 9.5.1; GraphPad Software, San Diego, CA, USA) at 5% probability. The LC_50_ values were calculated using Microsoft Excel (Microsoft Office 365, version 2408; Microsoft Corp., Redmond, WA, USA).

### 2.9. Docking Molecular and Molecular Dynamics

To investigate the binding modes of each compound isolated from technical CNSL, docking molecular and molecular dynamics simulations were performed following methodologies described in previous studies [28,29,30,31]. The structure of the monomeric form of AChE was obtained from the X-ray structure of *rh*AChE in complex with donepezil (PDB ID: 4EY7) [32]. The structure of compounds C1, C2 and C3 were optimized using Gaussian 09 software [33] at the B3LYP/6-31G(d,p) level of theory [34]. Their charges and electrostatic surface potentials (ESP) were computed at the HF/6-31G(d,p), and Antechamber [35] was used to parametrize all three substrates. The XYZ coordinates of the optimized structures, along with the parameter files containing atomic charges, angles, and dihedral terms derived from electrostatic potential (ESP) calculations, are provided in the Supporting Information.

AutoDock4 software [36] was used to explore the binding poses of the substrates to AChE active site. In the docking molecular protocol, the structure of the enzyme was kept fixed, while the substrate was flexible. A total of 100 poses were obtained for each protein complex. Cluster analysis was performed in the obtained poses and the structure with the highest binding affinity was chosen as the starting point of the all-atom molecular dynamics (MD) simulations. To validate the docking protocol, we carried out a redocking of donepezil to the AChE binding site. In this procedure, the ligand superimposed the crystal structure within a RMSD of 2 Å.

The classical MD simulations were performed with the PMEMD module from Amber22 software package [35]. The AMBER ff14SB force field was employed for the protein while the gaff2 force field was used for the substrates. The enzyme–substrate complex was then placed in a cubic box of 100 × 100 × 100 Å dimensions. The shortest distance from the edge of the box to the surface of the complex was not shorter than 10 Å. The TIP3P water model was used as the solvent, and Na^+^ and Cl^−^ ions were added to neutralize the total charge of the system, and cause a concentration of 0.154 M. After the initial minimization, the system was heated up to 300 K over 10 ns at constant volume (NVT), while imposing positional restraints of 100 kcal/mol. Å^2^ on the heavy atoms. Subsequently, restraints were slowly removed, and 100 ns of MD were performed using the isothermal-isobaric ensemble (NPT). The temperature control (300 K) was performed by a Langevin thermostat with a collision frequency of 1 ps^−1^, and the pressure control (1 atm) was accomplished by a Monte Carlo barostat. The SHAKE algorithm was used to constrain the bonds involving hydrogen atoms, and the particle mesh Ewald method was used to compute the electrostatic interactions, which for both Coulombic and Van der Waals interactions, a 12 Å cutoff distance was maintained. The trajectories were computed for with a time step of 1 fs. For each complex, the binding free energy was calculated by the MM/PBSA [37] and MM/GBSA [38] approaches based on the final 10 ns of simulation.

## 3. Results and Discussion

### 3.1. Chromatographic Analysis

**Figure 1:**
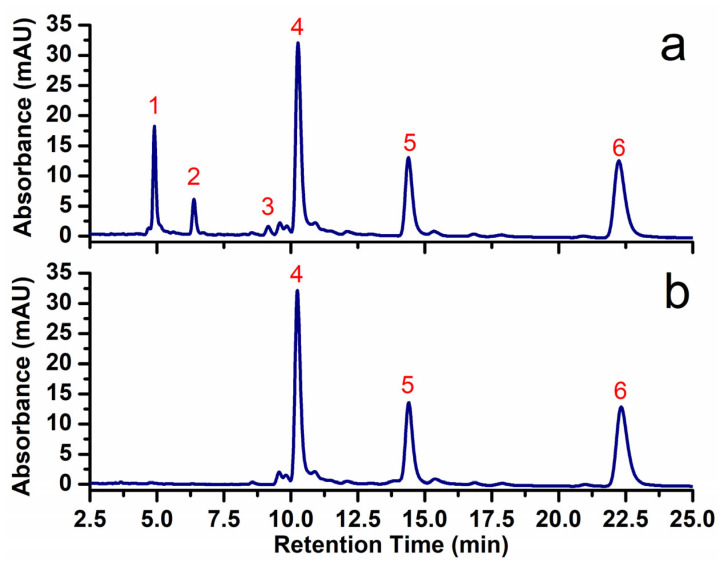
Representative high-performance liquid chromatography profile of: (**a**) technical CNSL constituents: peak 1-cardol (C_15:3_), peak 2-cardol (C_15:2_), peak 3-cardol (C_15:1_), peak 4-cardanol (C3), peak 5-cardanol (C2) and peak 6-cardanol (C1). (**b**) Cardanol mixture: peaks 4-cardanol triene (C3), 5-cardanol diene (C2), 6-cardanol monoene (C1). Column: Hypersil GOLD 25 cm, running time: 25 min, flow rate: 1.80 mL/min, mobile phase: 80% acetonitrile and 20% of a 1% (*v*/*v*) aqueous acetic acid solution.

The composition of cashew nutshell liquid (CNSL) is strongly influenced by the extraction method employed. The HPLC chromatogram of technical CNSL, shown in (Figure 1a), revealed six distinct peaks: peaks 1, 2, and 3, which correspond to tri-, di-, and monounsaturated cardol compounds, while peaks 4, 5, and 6, represent the tri- (C3), di- (C2), and monounsaturated (C1) cardanol derivatives. Notably, cardanol was identified as the most abundant constituent among the alkylphenol compounds in the analyzed CNSL sample. The retention times and relative percentage recoveries of each constituent are summarized in Table 1.

The mixture of cardanols obtained by solvent extraction with hexane from technical CNSL is shown in Figure 1b. The resulting chromatogram displayed only peaks 4, 5, and 6, confirming the selective extraction of cardanol, since the cardol-associated peaks (1, 2, and 3) were absent. As expected, no anacardic acid peaks were detected in the chromatogram of thermally treated CNSL, confirming that anacardic acids underwent decarboxylation and were converted into their corresponding cardanol derivatives [7,12,15].

The relative percentage recoveries for C1, C2, and C3 was 29%, 20%, and 37%, respectively. The analyzed peaks accounted for a total yield of 86% of the compounds present in cardanol. These findings align with previous reports, which successfully isolated three cardanol components: monoene (42%), diene (22%), and triene (36%), with high purity [39].

### 3.2. Nuclear Magnetic Resonance and Infrared Spectroscopy

**Figure 2:**
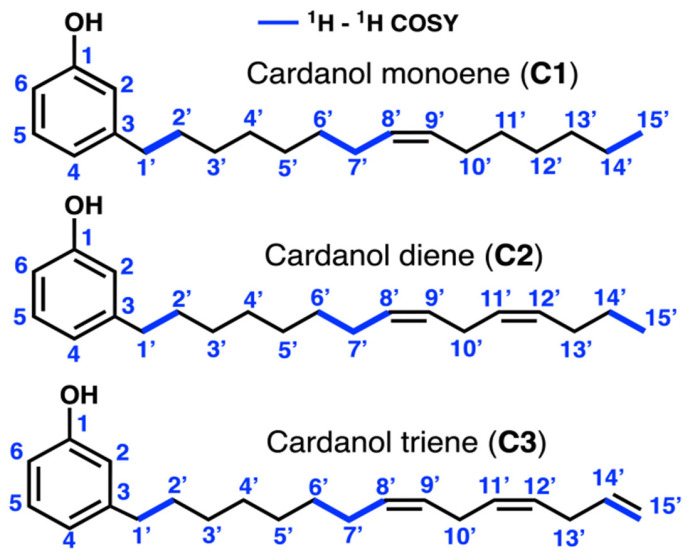
Selected COSY (bold blue lines) correlations and atom numbering of cardanol compounds present in the CNSL.

A detailed ^1^H and ^13^C NMR characterization with complete and unambiguous assignments for all carbon and hydrogen atoms of the three main cardanol components in CNSL is presented in Table 2. The ^1^H NMR spectra of all three cardanol compounds display aromatic proton signals (H1–H6) within the δ 6.6–7.14 range. Among these, the least shielded signal is observed at H3 (δ 7.1, t, *J* = 7.5 Hz), corresponding to the proton in the meta position relative to the hydroxyl group. The presence of unsaturated double bonds in the alkyl chain is evident in the monoene cardanol, where the vinylic protons H8′-H9′ resonate as a multiplet at δ 5.35–5.39. In the diene cardanol, the main difference is the increased integration of the multiplet located at δ 5.32–5.43, which corresponds to four hydrogen atoms (H8′, H9′, H11′, and H12′). In the triene compound, two additional peaks appear at δ 4.98–5.50 (H15′) and δ 5.77–5.90 (H14′), associated with terminal vinyl unsaturation. The remaining proton signals, corresponding to the long alkyl chain, are observed in the upfield region between δ 3.0–0.8. The ^1^H–^1^H COSY (Figure 2) data further confirmed the attachment of the alkyl chain to the phenyl ring, as well as the specific locations of the double bonds within the chain, based on correlations between H14′ and H15′, H7′ and its neighboring protons H6′ and H8′, and H2′ and H1′.

**Figure 3:**
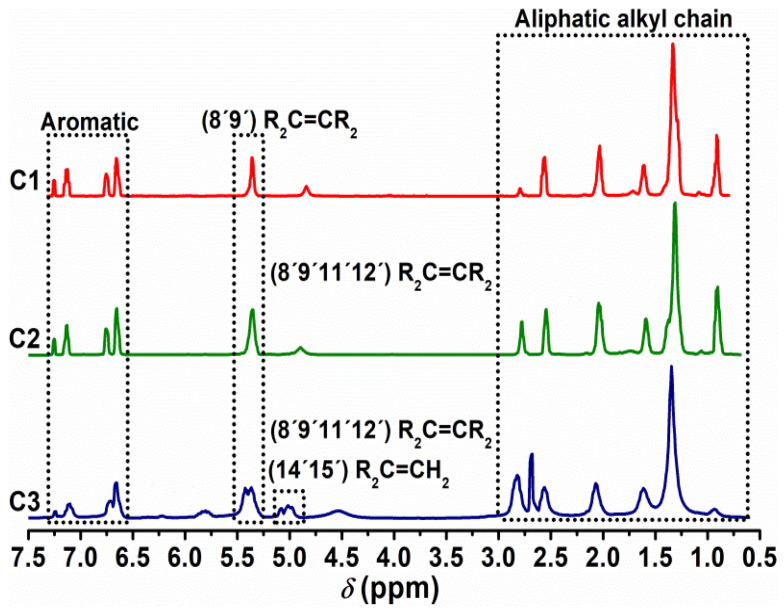
^1^H NMR (500 MHz) spectra of cardanol mono- (C1), di- (C2), and triene (C3) compounds present in the CNSL, recorded in CDCl_3_.

The ^13^C NMR spectra (Figure 3), complemented by HSQC, provided detailed assignments for both aromatic and aliphatic carbon atoms. The characteristic unsaturated carbon atoms appeared within the range of δ 155–112, with the signal at δ 155 corresponding to the aromatic carbon bound to the hydroxyl group (C1). The remaining aromatic carbons displayed chemical shifts consistent with substituted benzene systems, with C2 and C3 appearing at δ 112.69 and δ 145.14, respectively. The aliphatic carbon signals, corresponding to the alkyl chain, were observed within the δ 14–32 range, characteristic of long hydrocarbon chains. The olefinic carbon signals for the unsaturated side chains were found at δ 130.06 and δ 129.59 for the monoene, δ 130.06 and δ 128.22 for the diene, and δ 130.61, δ 127.80, and δ 127.05 for the triene. Notably, the terminal vinyl carbons in the triene compound were assigned to δ 137.04 (C14′) and δ 114.90 (C15′), confirming the presence of conjugated diene and triene systems. The chemical shifts of C1′ (δ 36.04) and C2′ (δ 31.49) were consistent with benzylic and adjacent methylene carbons, respectively. The downfield shift of C10′ (δ 32.00) in the diene compound compared to the monoene (δ 27.41) further supports the introduction of additional unsaturation. 

**Figure 4:**
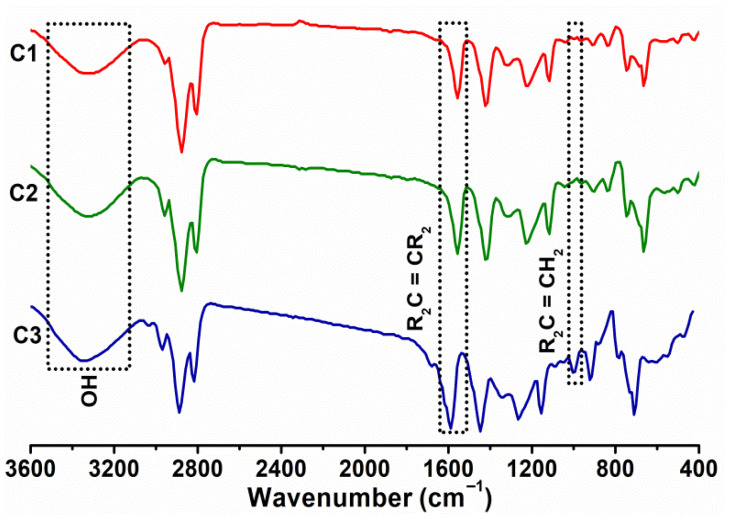
Infrared spectra of cardanol mono- (C1), di- (C2) and triene (C3) compounds present in the CNSL.

The infrared absorption spectra of the cardanol compounds extracted from CNSL exhibit distinct vibrational bands corresponding to their functional groups (Figure 4). The broad absorption band at 3339 cm^−1^, along with a weaker signal at 1347 cm^−1^, corresponds to the stretching and bending vibrations of the phenolic hydroxyl (-OH) group, respectively. The presence of unsaturation within the alkyl chain is indicated by the C-H stretching vibration of the inner double bond, observed at 3009 cm^−1^. The characteristic asymmetric and symmetric stretching vibrations of methyl, methylene, and methine groups appear at 2923 and 2852 cm^−1^, respectively.

The aromatic ring exhibits characteristic C=C stretching vibrations at 1609 and 1581 cm^−1^ for the symmetric mode, while the asymmetric stretches appear at 1261 and 1149 cm^−1^. Additionally, the vibrational bands at 778 and 691 cm^−1^ are attributed to the out-of-plane bending of hydrogen atoms adjacent to the benzene ring, further confirming the presence of the alkylphenol structure [10,40,41].

A notable distinction is observed among the cardanol derivatives in the bands at 990 and 910 cm^−1^, which exhibit increased intensity in the triene compound. These bands correspond to the out-of-plane deformation of the terminal vinyl group (RCH=CH_2_) in the alkyl chain, highlighting the influence of the degree of unsaturation on the spectroscopic profile of cardanol [10,39].

### 3.3. Biological Activity of Cardanols from CNSL

Table 3 presents a summary of the biological activity results of cardanols derived from CNSL. The evaluated parameters include DPPH radical scavenging capacity, AChE inhibition activity, lethality towards brine shrimp (*Artemia salina*).

The antioxidant activity of the cardanols from CNSL was evaluated using the DPPH radical scavenging assay. As observed in the chromatographic analysis, the relative polarity of the cardanol compounds increases with the degree of unsaturation, a trend also reflected in their antioxidant capacity. Statistical analysis of the DPPH radical scavenging data revealed that the triene (C3) and diene (C2) compounds exhibited significantly higher antioxidant activity than the monounsaturated cardanol (C1). Specifically, compounds C3 and C2 scavenged DPPH radicals at concentrations approximately three times lower than C1.

**Table 3:** Results of the DPPH radical scavenging assay, Brine Shrimp Lethality Test (BSLT), and acetylcholinesterase (AChE) inhibition assay for the cardanol mixture (Cardanol) and its main constituents: cardanol monoene (C1), cardanol diene (C2), and cardanol triene (C3).

Compound	DPPH IC_50_ (µg/mL, 95% CI)	DPPH IC_50_ (µM, 95% CI)	BSLT LC_50_ (µg/mL, 95% CI)	AChE Inhibition Zone (cm)
C1	1000.00 ± 200.00 ^a^ [773.68–1226.32]	3311.26 ± 662.25 [2561.88–4060.12]	43,186.00 ± 1991.00 ^a^ [40,932.97–45,439.03]	0.6
C2	340.00 ± 20.00 ^b^ [317.37–362.63]	1133.33 ± 67.00 [1057.18–1208.82]	41,973.00 ± 1991.00 ^a^ [39,719.97–44,226.03]	0.6
C3	179.00 ± 5.00 ^c^ [173.34–184.66]	600.00 ± 17.00 [580.76–619.24]	4118.00 ± 328.00 ^b^ [3746.83–4489.17]	0.8
Cardanol	551.00 ± 20.00 ^b^ [528.37–573.63]	-	4270.00 ± 145.00 ^b^ [4105.92–4434.08]	0.9
Quercetin	4.77 ± 0.50	-		-

^a^, Significantly different from compounds marked with b and c for DPPH IC_50_ and BSLT LC_50_ results. ^b^, Significantly different from compounds marked with a and c for DPPH IC_50_ and BSLT LC_50_ results. ^c^, Significantly different from compounds marked with a and b for DPPH IC_50_ and BSLT LC_50_ results. a,b,c: Different superscript letters within the same column indicate statistically significant differences among samples (*p* < 0.05). Values are expressed as mean ± standard deviation (SD, *n* = 3). Statistical analyses were performed using one-way ANOVA followed by Tukey’s HSD post hoc test.

These results suggest a direct connection between the degree of unsaturation and increased antioxidant/AChE inhibitory activity in cardanols, which is supported by recent reports on natural and synthetic phenolic derivatives [20,21]. Reinforcing that cardanol derivatives are essential and sustainable phenolic groups for the development of multifunctional agents that combat oxidative stress and cholinergic dysfunction. Thus, cardanol has emerged as a potent antioxidant agent, consistent with previous reports [42], due to the presence of phenolic groups with a high capacity to scavenge free radicals. This can be attributed mainly to the phenolic hydroxyl groups of cardanol, which act efficiently in the capture of DPPH radicals through an electron transfer mechanism coupled to proton transfer [43,44]. Paula et al. further demonstrated the antioxidant potential of a mixture of saturated, monoene, diene, and triene cardanols derived from CNSL in naphthenic mineral oil, where cardanol addition enhanced oxidative stability by a factor of 4 to 5 [11]. Similarly, other CNSL constituents, including anacardic acids, cardanols, and cardols, exhibited antioxidant activity via DPPH and ABTS free radical scavenging assays [8]. Furthermore, previous studies by our research group demonstrated that cardanol nanoparticles (NPs) synthesized using polysaccharide-based systems (chitosan, sodium alginate, or gum arabic) exhibited enhanced antioxidant potential [10].

In the AChE inhibition assay, the cardanol mixture displayed an inhibition zone of 0.9 ± 0.1 cm, comparable to the standard inhibitor physostigmine (0.9 ± 0.1 cm). Among the individual constituents, C3 showed the highest inhibition (0.8 ± 0.1 cm), followed by C2 (0.6 ± 0.1 cm) and C1 (0.6 ± 0.1 cm). This inhibition trend was corroborated by enzyme-linked immunosorbent assay (ELISA) measurements, where C3 exhibited the strongest AChE inhibition [42].

The efficiency of antioxidant activity and affinity for AChE can be directly influenced by variations in solubility and lipophilicity of cardanols with different degrees of unsaturation, as described for long phenols of natural origin [8,19,42,45,46,47]. C3 indeed presented the shortest retention time in HPLC (indicating greater relative polarity, conformational flexibility, and lower lipophilicity), benefiting both the scavenging of DPPH radicals in methanolic media and deep hydrophobic interactions in the catalytic site of the enzyme [43,44,46].

However, the increased lipophilicity may also contribute to the greater toxicity observed against *A. salina*, a result consistent with previous data on the ecotoxicity of cardanols and cardols [9,16]. The lethality assay using brine shrimp (*Artemia* sp.) was employed to assess the toxicity of these constituents. As it is a robust and low-cost method, it has become an effective classification for large-scale analysis of industrial effluents [16]. The toxicity against *A. salina*, presented in Table 3, is demonstrated by the LC50, which is the concentration that inhibits 50% of the nauplii of *A. salina*. Lower numbers, LC50, correspond to greater bioactivity, and C3 and the cardanol mixture were the most bioactive, followed by C2 and C1. Therefore, the similarity between C3 and the cardanol mixture was already predicted, since C3 is the most abundant cardanol derivative. The activities of the compounds manifest themselves as toxicity to shrimp.

Different superscript letters in Table 3 indicate statistically significant differences among samples (*p* < 0.05).

The lethality to *A. salina* reported here represents a preliminary toxicity screening commonly used for CNSL-derived phenolics and should not be extrapolated to mammalian systems. Because mammalian cytotoxicity (CC_50_) was not determined in this study, a selectivity index (CC_50_/AChE IC_50_) could not be calculated. Future studies will address CC_50_ in mammalian cell lines to better contextualize the safety window of the most active cardanol (C3).

### 3.4. Docking Molecular and Molecular Dynamics Simulations

**Figure 5:**
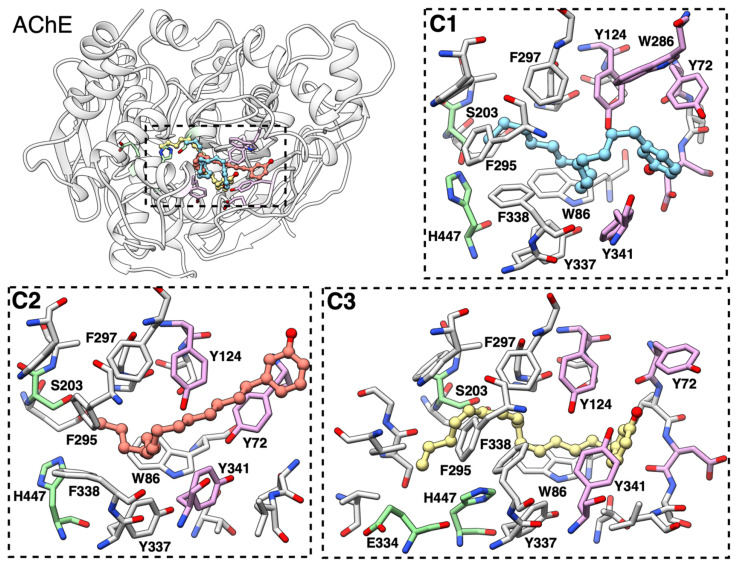
Lowest energy binding configurations of cardanol mono- (C1), di- (C2) and triene (C3) compounds inside the active site of acetylcholinesterase (AChE). Residues of the catalytic active site are shown in green and residues of the peripherical anionic site are shown in purple.

Natural cardanol compounds derived from cashew nutshell liquid (CNSL) have been identified as promising scaffolds for developing novel inhibitors with enhanced anti-amyloid and antioxidant activities. To elucidate their molecular interactions with acetylcholinesterase (AChE), docking molecular and molecular dynamics (MD) simulations were performed, revealing the preferential binding modes of compounds C1, C2, and C3 (Figure 5 and Figure S1). All three compounds exhibited suitable conformational flexibility, spanning both the peripheral anionic site (PAS) and the catalytic active site (CAS). In most docking poses, the unsaturated alkyl side chain was oriented toward the buried CAS, while the phenol ring remained solvent-exposed near the PAS. This orientation favored extensive hydrophobic interactions but precluded hydrogen bonding, as the CAS is deeply buried and the phenolic hydroxyl (-OH) group remained exposed to the solvent.

Binding affinity calculations using both MMGBSA and MMPBSA methods confirmed that C3 exhibited the highest affinity for AChE, with MMGBSA binding free energies of C1 = −6.4 ± 1.3 kcal/mol, C2 = −7.6 ± 1.9 kcal/mol, and C3 = −10.6 ± 2.3 kcal/mol, and MMPBSA values of C1 = −8.1 ± 2.0 kcal/mol, C2 = −9.3 ± 2.1 kcal/mol, and C3 = −13.4 ± 2.5 kcal/mol. Average contribution of each residue towards the total binding energy is shown in Table S1. These values are comparable to those reported in a previous study (cite: Molecular docking analysis of acetylcholinesterase inhibitors for Alzheimer’s disease) management indicating that the binding affinities observed here are within a similar energetic range. It is noteworthy, however, that the compounds analyzed in the present work are natural and unmodified, whereas those in the referenced study correspond to synthetically optimized drug molecules, which may account for subtle differences in the interaction profiles and overall binding energies.

The residue-specific interaction analysis revealed that C1 formed hydrophobic contacts with PAS residues Y72, Y124, W286, Y341, and F295, while interacting within the CAS with H447, F338, S203, W86, and F297. C2 exhibited a similar binding pattern but with slightly stronger interactions in the CAS, particularly with Y337, H447, F338, S203, and W86, contributing to its higher binding affinity relative to C1. C3 demonstrated the strongest binding affinity, stabilizing its position in the CAS through interactions with H447, E334, Y337, W86, F338, S293, and F297, while retaining PAS contacts with Y72, Y124, Y341, and F295. And this can be due in part to the differences in polarity of compounds: C3 > C2 > C1, as shown by the HPLC retention times.

Given the hydrophobic nature of these compounds, their binding mechanism was predominantly mediated by van der Waals forces and π–π stacking interactions rather than hydrogen bonding. Unlike polar inhibitors that engage in electrostatic interactions with S203, E334, or H447, the alkyl chains of C1, C2, and C3 favored deep burial in the CAS, contributing to their stabilization within the active site. The solvent-exposed phenolic hydroxyl (-OH) group, while capable of hydrogen bonding in aqueous environments, did not directly participate in enzyme interactions. The stronger binding affinity observed for C3 can be attributed to a greater number of hydrophobic interactions within the CAS, particularly with E334, which further stabilized its alkyl chain compared to C1 and C2. Additionally, interactions with Y337 and W86, conserved across all three compounds, reinforced their anchoring across both PAS and CAS, ensuring an optimal binding orientation.

Preferential double-bond interactions are of interest for the development of next-generation AChE inhibitors with potential antioxidant capacity. To enhance interactions with the catalytic active site (CAS) of acetylcholinesterase (AChE), chemical modifications of cardanol derivatives have been explored by another research group. Notably, the introduction of a terminal hydroxyl (-OH) group to the hydrophobic alkyl chain has been shown to improve solubility and membrane permeability, facilitating additional hydrogen bonding interactions within the CAS. This modification enhanced target recognition and binding affinity, as evidenced by the increased inhibitory potency of the modified compounds [45,46,47].

## 4. Conclusions

In this study, cardanol was successfully extracted and purified from technical cashew nutshell liquid (CNSL), yielding a viscous, dark oil with 87% purity. Among its main constituents, 3-(n-pentadec-8-enyl) phenol, 3-(n-penta-8,11-dienyl) phenol, and 3-(n-pentadeca-8,11,14-trienyl) phenol, a clear trend was observed in which increased unsaturation correlated with enhanced biological activity, including stronger free radical scavenging, higher acetylcholinesterase (AChE) inhibition, and greater lethality against *Artemia salina*. These findings underscore the relevance of unsaturation degree in modulating bioactivity.

Although the brine shrimp lethality test (BSLT) provided a useful preliminary indication of the general toxicity of the cardanol derivatives, these data should be interpreted only as a screening reference and not as a predictor of mammalian cytotoxicity. Future studies will determine the cytotoxic concentration (CC_50_) in relevant mammalian cell lines in order to calculate the selectivity index (SI = CC_50_/AChE IC_50_) and better assess the safety margin of the most active compound (C3).

Complementary molecular docking and molecular dynamics simulations revealed key interactions within AChE’s active and peripheral sites, supporting the experimental structure-activity relationships and providing mechanistic insight into the binding behavior of these phenolic compounds. Taken together, the results not only provide a mechanistic understanding but also establish a sustainable chemical space. The in vitro and in silico results of cardanol derivatives are encouraging; however, further investigation into their pharmacokinetic properties and in vivo efficacy will be essential to demonstrate their therapeutic potential for drug discovery applications in Alzheimer’s disease and related neurodegenerative conditions.

## Figures and Tables

**Table 1:** High-performance liquid chromatography analysis of technical cashew nutshell liquid.

Peak Number	Constituent	Retention Time (min)	Yield (%)
1	Cardol triene	4.59	10.39
2	Cardol diene	6.10	3.57
3	Cardol monoene	8.93	1.16
4	Cardanol triene	10.06	36.91
5	Cardanol diene	14.26	20.25
6	Cardanol monoene	22.27	29.28

**Table 2:** ^1^H (500 MHz) and 13C (125 MHz) NMR data for Cardanol mono-, di-, and triene compounds present in CNSL, recorded in CDCl_3_.

Position	Cardanol Monoene (C1)		Cardanol Diene (C2)		Cardanol Triene (C3)	
	d_C_, Type	d_H_, (*J* in Hz)	d_C_, Type	d_H_, (*J* in Hz)	d_c_, Type	d_H_, (*J* in Hz)
1	155.66, C	-	155.65, C	-	155.95, C	-
2	112.69, C	-	112.69, C	-	112.77, C	-
3	145.14, C	-	145.12, C	-	145.02, C	-
4	121.16, CH	6.66, d (10.0)	121.15, CH	6.66, d (10.0)	120.89, CH	6.67, d (7.5)
5	130.36, CH	7.14, t (7.5)	130.35, CH	7.15, t (7.5)	137.04, CH	7.13, t (7.5)
6	115.52, CH	6.76, d (10.0)	115.51, CH	6.77, d (5.0)	115.58, CH	6.74, d (7.5)
1′	36.04, CH_2_	2.57, t (7.5)	36.02, CH_2_	2.56, q (7.5)	36.04, CH_2_	2.58, t (7.5)
2′	31.49, CH_2_	1.61, q (5.0)	31.48, CH_2_	1.60, q (5.0)	31.46, CH_2_	1.59, q (6.0)
3′, 4′ 5′, 6′	29.21–29.96, CH_2_	1.27–1.41	29.20–29.95, CH_2_	1.27–1.42	29.42–29.82, CH_2_	1.33 (8.0)
7′	27.45, CH_2_	2.03, bq (12.5)	27.43, CH_2_	2.05, bq (10.0)	27.43, CH_2_	2.06, bq (6.0)
8′	130.06, CH	5.38, bt (6.0)	130.06, CH	5.32–5.43, m	130.61, CH	5.31–5.50, m
9′	129.59, CH	5.36, bq (6.0)	130.06, CH	5.32–5.43, m	130.61, CH	5.31–5.50, m
10′	27.41, CH_2_	2.03, m	32.00, CH_2_	2.79, t (5.0)	31.76, CH_2_	2.82, m
11′	29.21–29.96, CH_2_	1.27–1.41, m	128.22, CH	5.32–5.43, m	127.80–127.05, CH	5.31–5.50, m
12′	29.21–29.96, CH_2_	1.27–1.41, m	128.22, CH	5.32–5.43, m	127.80–127.05, CH	5.31–5.50, m
13′	29.21–29.96, CH_2_	1.27–1.41, m	27.43, CH_2_	1.27–1.42, m	25.78, CH_2_	2.82, m
14′	29.21–29.96, CH_2_	1.27–1.41, m	29.20–29.95, CH_2_	1.27–1.42, m	137.04–114.90, CH	5.83, m
15′	14.02, CH_3_	0.91, t (15.0)	14.02, CH_3_	0.91, t (15.0)	137.04–114.90, CH_2_	5.07, dd (5.3) 4.99, d (9.0)

## Data Availability

No new data were created or analyzed in this study.

## Supplementary Materials

The following supporting information can be downloaded at: https://www.mdpi.com/article/10.3390/bioengineering12121316/s1, Computational Section. Table S1. Energy decomposition by the MM/GBSA method for cardanol compounds. Figure S1. Root mean square deviation (RMSD) plots.

## Abbreviations

The following abbreviations are used in this manuscript:

CIONE	Companhia Industrial de Óleos do Nordeste
BSLT	Brine Shrimp Lethality Test
CNSL	Cashew nutshell liquid
HPLC	High Performance Liquid Chromatography
AChE	Acetylcholinesterase
DPPH	1,1-diphenyl-2-picrylhydrazyl
DMSO	Dimethyl Sulfoxide
DTNB	5,5′-Dithiobis-(2-nitrobenzoic acid)
ACTI	Acetylthiocholine iodide
CAS	Catalytic Active Site
NMR	*Nuclear Magnetic Resonance*
PAS	Peripheral Catalytic Site
TLC	Thin-layer Chromatography
ESP	Electrostatic Potential
NPT	Isothermal-isobaric Ensemble
NVT	ns at constant volume
NPs	nanoparticles
MD	Molecular Dynamics
