# Supplementary material for: Unsaturation-Driven Modulation of Antioxidant and Acetylcholinesterase Inhibitory Activities of Cardanol Derivatives
Source: Bioengineering (Basel). 2025 Dec 1;12(12):1316. doi: 10.3390/bioengineering12121316 (PMC12729563; doi:10.3390/bioengineering12121316)
Supplement: Supplementary file 1 [file bioengineering-12-01316-s001.zip › bioengineering-3924586-supplementary.pdf]

bioengineering

MDPI

Article

Unsaturation-Driven Modulation of Antioxidant and Acetylcholinesterase Inhibitory Activities of Cardanol Derivatives

Roberta Bussons Rodrigues Valerio [1,2,3], Halisson de Souza [2], Vitor Martins [2], Katherine Silva [2], Jane Eire de Manezes [1], Anderson Chaves [3], Leonardo F. Serafim [4], Antônio Vieira-Neto [5], Jose Cleiton S. dos Santos [1,6,*] and Selene de Morais [1,2]

[1] Postgraduate Program in Natural Sciences, State University of Ceara—UECE, Itaperi Campus, 60714-903 Fortaleza, CE, Brazil; rbussons@hotmail.com (R.B.R.V.)
[2] Laboratory of Natural Products Chemistry, State University of Ceara—UECE, Itaperi Campus, 60714-903 Fortaleza, CE, Brazil
[3] Advanced Materials Chemistry Group (GQMAT), Department of Analytical and Physical Chemistry, Federal University of Ceara—UFC, Pici Campus, CP 12100, 60451-970 Fortaleza, CE, Brazil
[4] Department of Chemistry, Georgia State University, Atlanta, GA 30302, USA
[5] Center for Experimental Biology, University of Fortaleza, Av. Washington Soares, 1321, 60811-905 Fortaleza, CE, Brazil; aevneto@gmail.com
[6] Institute of Engineering and Sustainable Development (IEDS), University of International Integration of Afro-Brazilian Lusophony—UNILAB, Campus das Auroras, Rua Jose Franco de Oliveira, s/n, 62790-970 Redenc.o, CE, Brazil
* Correspondence: jcs@unilab.edu.br

SUPPLEMENTARY MATERIAL

Computational Section

C1 - XYZ coordinates (.pdb)

ATOM	1	O1	C11	1	-8.363	13.508	-3.621	-0.537993
ATOM	2	H1	C11	1	-7.984	13.541	-2.733	0.373908
ATOM	3	C9	C11	1	-9.278	12.479	-3.683	0.378438
ATOM	4	C10	C11	1	-9.030	11.243	-3.080	-0.364608
ATOM	5	H2	C11	1	-8.099	11.096	-2.534	0.145480
ATOM	6	C8	C11	1	-10.459	12.687	-4.399	-0.225557
ATOM	7	H3	C11	1	-10.639	13.654	-4.859	0.123818
ATOM	8	C7	C11	1	-11.384	11.648	-4.498	-0.040749
ATOM	9	H4	C11	1	-12.310	11.805	-5.046	0.090829
ATOM	10	C6	C11	1	-11.141	10.416	-3.888	-0.215378
ATOM	11	H5	C11	1	-11.886	9.626	-3.956	0.107793
ATOM	12	C5	C11	1	-9.954	10.194	-3.174	0.138386
ATOM	13	C4	C11	1	-9.663	8.864	-2.499	0.014878
ATOM	14	H6	C11	1	-10.588	8.525	-2.016	-0.009517
ATOM	15	H7	C11	1	-8.950	9.063	-1.691	-0.009517
ATOM	16	C3	C11	1	-9.063	7.675	-3.345	0.095762
ATOM	17	H8	C11	1	-9.518	6.757	-2.962	-0.019465
ATOM	18	H9	C11	1	-7.998	7.608	-3.093	-0.019465
ATOM	19	C2	C11	1	-9.187	7.727	-4.894	-0.015598
ATOM	20	H10	C11	1	-8.302	8.247	-5.276	-0.000735
ATOM	21	H11	C11	1	-10.034	8.355	-5.174	-0.000735
ATOM	22	C1	C11	1	-9.291	6.381	-5.687	-0.071409
ATOM	23	H12	C11	1	-8.283	6.086	-5.989	0.022095
ATOM	24	H13	C11	1	-9.822	6.627	-6.613	0.022095
ATOM	25	C11	C11	1	-10.025	5.169	-5.032	0.006380
ATOM	26	H14	C11	1	-10.101	5.302	-3.947	0.009381
ATOM	27	H15	C11	1	-11.055	5.159	-5.407	0.009381
ATOM	28	C12	C11	1	-9.445	3.746	-5.300	-0.077457
ATOM	29	H16	C11	1	-9.821	3.077	-4.518	0.000541
ATOM	30	H17	C11	1	-9.843	3.364	-6.249	0.000541
ATOM	31	C13	C11	1	-7.900	3.603	-5.337	0.463182
ATOM	32	H18	C11	1	-7.455	4.121	-4.477	-0.095857
ATOM	33	H19	C11	1	-7.518	4.091	-6.239	-0.095857
ATOM	34	C14	C11	1	-7.453	2.155	-5.376	-0.362524
ATOM	35	H20	C11	1	-7.146	1.788	-6.357	0.116588
ATOM	36	C15	C11	1	-7.412	1.267	-4.374	-0.124057
ATOM	37	H21	C11	1	-7.098	0.257	-4.644	0.079245
ATOM	38	C16	C11	1	-7.734	1.472	-2.904	0.120284
ATOM	39	H22	C11	1	-7.943	0.486	-2.477	-0.022085
ATOM	40	H23	C11	1	-8.654	2.061	-2.798	-0.022085
ATOM	41	C17	C11	1	-6.584	2.137	-2.070	0.058837

ATOM	42	H24	C11	1	-5.681	2.164	-2.688	-0.020894
ATOM	43	H25	C11	1	-6.359	1.493	-1.211	-0.020894
ATOM	44	C18	C11	1	-6.939	3.554	-1.548	0.075111
ATOM	45	H26	C11	1	-7.578	4.039	-2.295	-0.026962
ATOM	46	H27	C11	1	-7.563	3.429	-0.656	-0.026962
ATOM	47	C19	C11	1	-5.775	4.547	-1.173	-0.081932
ATOM	48	H28	C11	1	-5.916	5.458	-1.766	0.014102
ATOM	49	H29	C11	1	-5.926	4.845	-0.131	0.014102
ATOM	50	C20	C11	1	-4.287	4.090	-1.320	0.133978
ATOM	51	H30	C11	1	-4.212	3.302	-2.074	-0.025408
ATOM	52	H31	C11	1	-3.718	4.941	-1.707	-0.025408
ATOM	53	C21	C11	1	-3.603	3.569	-0.013	-0.155056
ATOM	54	H32	C11	1	-2.632	4.047	0.153	0.033011
ATOM	55	H33	C11	1	-4.217	3.768	0.871	0.033011
ATOM	56	H34	C11	1	-3.426	2.489	-0.049	0.033011

Charge file for AMBER (.prepin)

1	DUMM	DU	M	0	-1	-2	0.000	.0	.0	.00000
2	DUMM	DU	M	1	0	-1	1.449	.0	.0	.00000
3	DUMM	DU	M	2	1	0	1.523	111.21	.0	.00000
4	O1	oh	M	3	2	1	1.540	111.208	-180.000	-0.537993
5	H1	ho	E	4	3	2	0.966	113.099	-92.128	0.373908
6	C9	ca	M	4	3	2	1.378	48.408	3.448	0.378438
7	C10	ca	S	6	4	3	1.397	121.539	-145.404	-0.364608
8	H2	ha	E	7	6	4	1.089	119.166	1.063	0.145480
9	C8	ca	M	6	4	3	1.397	118.242	36.231	-0.225557
10	H3	ha	E	9	6	4	1.086	119.338	-1.824	0.123818
11	C7	ca	M	9	6	4	1.395	119.097	178.605	-0.040749
12	H4	ha	E	11	9	6	1.087	119.540	179.441	0.090829
13	C6	ca	M	11	9	6	1.396	120.730	0.230	-0.215378
14	H5	ha	E	13	11	9	1.088	119.622	178.493	0.107793
15	C5	ca	M	13	11	9	1.403	120.618	-0.654	0.138386
16	C4	c3	M	15	13	11	1.520	121.776	179.951	0.014878
17	H6	hc	E	16	15	13	1.097	107.729	-42.106	-0.009517
18	H7	hc	E	16	15	13	1.096	107.049	-156.033	-0.009517
19	C3	c3	M	16	15	13	1.578	119.617	82.838	0.095762
20	H8	hc	E	19	16	15	1.094	106.650	-143.418	-0.019465
21	H9	hc	E	19	16	15	1.096	106.989	103.719	-0.019465
22	C2	c3	M	19	16	15	1.555	118.597	-18.552	-0.015598
23	H10	hc	E	22	19	16	1.095	107.392	89.551	-0.000735
24	H11	hc	E	22	19	16	1.091	109.686	-24.433	-0.000735
25	C1	c3	M	22	19	16	1.566	118.760	-149.598	-0.071409
26	H12	hc	E	25	22	19	1.093	108.105	-92.972	0.022095
27	H13	hc	E	25	22	19	1.095	105.503	154.074	0.022095

28	C11	c3	M	25	22	19	1.561	119.091	33.281	0.006380
29	H14	hc	E	28	25	22	1.096	110.723	-18.890	0.009381
30	H15	hc	E	28	25	22	1.096	107.780	96.560	0.009381
31	C12	c3	M	28	25	22	1.560	117.476	-143.382	-0.077457
32	H16	hc	E	31	28	25	1.096	107.866	159.977	0.000541
33	H17	hc	E	31	28	25	1.098	109.341	-84.868	0.000541
34	C13	c3	M	31	28	25	1.552	117.277	38.400	0.463182
35	H18	hc	E	34	31	28	1.098	109.953	46.924	-0.095857
36	H19	hc	E	34	31	28	1.094	109.028	-70.186	-0.095857
37	C14	c2	M	34	31	28	1.516	112.467	170.599	-0.362524
38	H20	ha	E	37	34	31	1.091	115.293	101.679	0.116588
39	C15	c2	M	37	34	31	1.339	128.537	-76.856	-0.124057
40	H21	ha	E	39	37	34	1.092	115.923	177.108	0.079245
41	C16	c3	M	39	37	34	1.519	128.907	-3.360	0.120284
42	H22	hc	E	41	39	37	1.095	107.245	159.677	-0.022085
43	H23	hc	E	41	39	37	1.098	110.099	44.075	-0.022085
44	C17	c3	M	41	39	37	1.569	114.609	-80.036	0.058837
45	H24	hc	E	44	41	39	1.095	108.367	-10.174	-0.020894
46	H25	hc	E	44	41	39	1.097	108.534	-125.687	-0.020894
47	C18	c3	M	44	41	39	1.551	113.479	113.432	0.075111
48	H26	hc	E	47	44	41	1.096	107.954	-33.812	-0.026962
49	H27	hc	E	47	44	41	1.096	107.461	79.956	-0.026962
50	C19	c3	M	47	44	41	1.575	119.131	-157.766	-0.081932
51	H28	hc	E	50	47	44	1.096	107.462	123.550	0.014102
52	H29	hc	E	50	47	44	1.094	107.241	-123.174	0.014102
53	C20	c3	M	50	47	44	1.564	119.775	-0.491	0.133978
54	H30	hc	E	53	50	47	1.093	109.927	25.403	-0.025408
55	H31	hc	E	53	50	47	1.094	107.504	140.723	-0.025408
56	C21	c3	M	53	50	47	1.564	115.778	-97.059	-0.155056
57	H32	hc	E	56	53	50	1.095	111.654	-130.329	0.033011
58	H33	hc	E	56	53	50	1.095	111.649	-10.341	0.033011
59	H34	hc	E	56	53	50	1.095	111.818	109.829	0.033011

C2 - XYZ coordinates (.pdb)

ATOM	1	O1	C22	1	-31.140	-2.426	13.901	-0.572213
ATOM	2	H13	C22	1	-30.577	-3.133	14.241	0.411603
ATOM	3	C9	C22	1	-32.337	-2.962	13.509	0.403399
ATOM	4	C10	C22	1	-32.622	-4.326	13.629	-0.389404
ATOM	5	H12	C22	1	-31.872	-4.993	14.050	0.123984
ATOM	6	C8	C22	1	-33.287	-2.092	12.969	-0.240100
ATOM	7	H11	C22	1	-33.049	-1.038	12.878	0.140144
ATOM	8	C7	C22	1	-34.515	-2.605	12.561	-0.042878
ATOM	9	H10	C22	1	-35.260	-1.934	12.144	0.089760
ATOM	10	C6	C22	1	-34.803	-3.966	12.680	-0.249222

ATOM	11	H9	C22	1	-35.773	-4.336	12.365	0.119136
ATOM	12	C5	C22	1	-33.855	-4.847	13.216	0.202951
ATOM	13	C4	C22	1	-34.150	-6.324	13.404	-0.048243
ATOM	14	H7	C22	1	-34.829	-6.446	14.259	0.012629
ATOM	15	H8	C22	1	-33.224	-6.826	13.708	0.012629
ATOM	16	C3	C22	1	-34.724	-7.059	12.175	0.028993
ATOM	17	H5	C22	1	-35.282	-7.930	12.523	-0.001787
ATOM	18	H6	C22	1	-35.476	-6.432	11.688	-0.001787
ATOM	19	C2	C22	1	-33.655	-7.456	11.131	-0.005083
ATOM	20	H3	C22	1	-32.810	-6.765	11.226	-0.009052
ATOM	21	H4	C22	1	-34.051	-7.247	10.132	-0.009052
ATOM	22	C1	C22	1	-33.102	-8.915	11.129	0.059521
ATOM	23	H1	C22	1	-32.058	-8.863	10.798	-0.014139
ATOM	24	H2	C22	1	-33.604	-9.494	10.343	-0.014139
ATOM	25	C11	C22	1	-33.135	-9.722	12.436	-0.045668
ATOM	26	H14	C22	1	-32.401	-9.310	13.141	0.018538
ATOM	27	H15	C22	1	-34.101	-9.619	12.937	0.018538
ATOM	28	C12	C22	1	-32.841	-11.217	12.247	-0.063833
ATOM	29	H16	C22	1	-33.078	-11.758	13.170	0.008592
ATOM	30	H17	C22	1	-33.517	-11.626	11.483	0.008592
ATOM	31	C13	C22	1	-31.385	-11.527	11.849	0.249415
ATOM	32	H18	C22	1	-30.708	-11.017	12.541	-0.037806
ATOM	33	H19	C22	1	-31.188	-11.098	10.856	-0.037806
ATOM	34	C14	C22	1	-31.096	-13.001	11.812	-0.185777
ATOM	35	H20	C22	1	-31.659	-13.569	11.070	0.088761
ATOM	36	C15	C22	1	-30.247	-13.668	12.601	-0.313890
ATOM	37	H21	C22	1	-30.157	-14.743	12.463	0.114374
ATOM	38	C16	C22	1	-29.391	-13.054	13.693	0.459694
ATOM	39	H22	C22	1	-28.665	-13.805	14.032	-0.073722
ATOM	40	H23	C22	1	-28.782	-12.239	13.286	-0.073722
ATOM	41	C17	C22	1	-30.179	-12.574	14.888	-0.236463
ATOM	42	H24	C22	1	-30.933	-13.273	15.247	0.089559
ATOM	43	C18	C22	1	-30.044	-11.409	15.535	-0.190984
ATOM	44	H25	C22	1	-30.696	-11.240	16.390	0.097475
ATOM	45	C19	C22	1	-29.042	-10.321	15.247	0.104972
ATOM	46	H26	C22	1	-28.106	-10.569	15.768	-0.006403
ATOM	47	H27	C22	1	-28.765	-10.325	14.186	-0.006403
ATOM	48	C20	C22	1	-29.465	-8.887	15.663	0.092263
ATOM	49	H28	C22	1	-29.094	-8.685	16.673	-0.024769
ATOM	50	H29	C22	1	-28.917	-8.181	15.030	-0.024769
ATOM	51	C21	C22	1	-30.973	-8.566	15.579	-0.085506
ATOM	52	H30	C22	1	-31.543	-9.391	15.145	0.016366
ATOM	53	H31	C22	1	-31.401	-8.359	16.563	0.016366
ATOM	54	H32	C22	1	-31.161	-7.687	14.956	0.016366

Charge file for AMBER (.prepin)

1	DUMM	DU	M	0	-1	-2	0.000	.0	.0	.00000
2	DUMM	DU	M	1	0	-1	1.449	.0	.0	.00000
3	DUMM	DU	M	2	1	0	1.523	111.21	.0	.00000
4	O1	oh	M	3	2	1	1.540	111.208	-180.000	-0.572213
5	H13	ho	E	4	3	2	0.966	125.665	-25.683	0.411603
6	C9	ca	M	4	3	2	1.369	29.020	36.180	0.403399
7	C10	ca	S	6	4	3	1.399	122.378	-130.210	-0.389404
8	H12	ha	E	7	6	4	1.088	119.369	0.130	0.123984
9	C8	ca	M	6	4	3	1.397	117.488	50.018	-0.240100
10	H11	ha	E	9	6	4	1.084	119.248	0.085	0.140144
11	C7	ca	M	9	6	4	1.392	118.933	-179.971	-0.042878
12	H10	ha	E	11	9	6	1.086	119.346	179.752	0.089760
13	C6	ca	M	11	9	6	1.396	121.080	-0.221	-0.249222
14	H9	ha	E	13	11	9	1.085	119.480	179.257	0.119136
15	C5	ca	M	13	11	9	1.401	120.404	0.002	0.202951
16	C4	c3	M	15	13	11	1.518	121.862	177.894	-0.048243
17	H7	hc	E	16	15	13	1.099	108.940	-73.960	0.012629
18	H8	hc	E	16	15	13	1.096	108.407	172.540	0.012629
19	C3	c3	M	16	15	13	1.543	115.927	50.313	0.028993
20	H5	hc	E	19	16	15	1.091	108.447	-155.626	-0.001787
21	H6	hc	E	19	16	15	1.094	109.723	-42.216	-0.001787
22	C2	c3	M	19	16	15	1.546	113.766	79.899	-0.005083
23	H3	hc	E	22	19	16	1.096	108.225	-25.821	-0.009052
24	H4	hc	E	22	19	16	1.095	108.486	-137.779	-0.009052
25	C1	c3	M	22	19	16	1.560	119.080	98.539	0.059521
26	H1	hc	E	25	22	19	1.096	107.068	-147.855	-0.014139
27	H2	hc	E	25	22	19	1.098	109.393	99.874	-0.014139
28	C11	c3	M	25	22	19	1.536	118.845	-25.851	-0.045668
29	H14	hc	E	28	25	22	1.098	109.558	-70.444	0.018538
30	H15	hc	E	28	25	22	1.093	111.063	45.264	0.018538
31	C12	c3	M	28	25	22	1.535	113.742	168.015	-0.063833
32	H16	hc	E	31	28	25	1.096	109.611	-168.635	0.008592
33	H17	hc	E	31	28	25	1.099	109.278	-53.365	0.008592
34	C13	c3	M	31	28	25	1.541	114.119	69.039	0.249415
35	H18	hc	E	34	31	28	1.094	109.117	50.627	-0.037806
36	H19	hc	E	34	31	28	1.099	108.909	-64.856	-0.037806
37	C14	c2	M	34	31	28	1.503	112.673	174.037	-0.185777
38	H20	ha	E	37	34	31	1.091	115.357	63.482	0.088761
39	C15	c2	M	37	34	31	1.337	126.649	-115.419	-0.313890
40	H21	ha	E	39	37	34	1.088	118.080	179.040	0.114374
41	C16	c3	M	39	37	34	1.517	125.519	-0.572	0.459694
42	H22	hc	E	41	39	37	1.098	108.566	-169.233	-0.073722

43	H23	hc	E	41	39	37	1.096	110.316	-54.891	-0.073722
44	C17	c2	M	41	39	37	1.510	113.819	69.762	-0.236463
45	H24	ha	E	44	41	39	1.089	114.723	45.543	0.089559
46	C18	c2	M	44	41	39	1.339	127.320	-134.689	-0.190984
47	H25	ha	E	46	44	41	1.088	117.008	-179.204	0.097475
48	C19	c3	M	46	44	41	1.507	127.063	-2.089	0.104972
49	H26	hc	E	48	46	44	1.100	108.218	-84.707	-0.006403
50	H27	hc	E	48	46	44	1.097	110.503	28.606	-0.006403
51	C20	c3	M	48	46	44	1.552	115.766	153.607	0.092263
52	H28	hc	E	51	48	46	1.095	108.992	92.745	-0.024769
53	H29	hc	E	51	48	46	1.095	107.719	-154.819	-0.024769
54	C21	c3	M	51	48	46	1.544	116.342	-32.351	-0.085506
55	H30	hc	E	54	51	48	1.093	111.970	-4.955	0.016366
56	H31	hc	E	54	51	48	1.093	111.894	115.477	0.016366
57	H32	hc	E	54	51	48	1.094	111.467	-124.671	0.016366

C3 - XYZ coordinates (.pdb)

ATOM	1	O1	C33	1	-32.030	-1.374	14.225	-0.566139
ATOM	2	H13	C33	1	-31.575	-1.854	14.929	0.406955
ATOM	3	C9	C33	1	-32.917	-2.217	13.613	0.395426
ATOM	4	C10	C33	1	-33.095	-3.542	14.021	-0.364152
ATOM	5	H12	C33	1	-32.513	-3.930	14.854	0.119379
ATOM	6	C8	C33	1	-33.663	-1.702	12.548	-0.245240
ATOM	7	H11	C33	1	-33.514	-0.671	12.248	0.141465
ATOM	8	C7	C33	1	-34.584	-2.526	11.907	-0.040606
ATOM	9	H10	C33	1	-35.170	-2.129	11.084	0.088642
ATOM	10	C6	C33	1	-34.766	-3.850	12.312	-0.238173
ATOM	11	H9	C33	1	-35.495	-4.476	11.809	0.115187
ATOM	12	C5	C33	1	-34.017	-4.374	13.374	0.187428
ATOM	13	C4	C33	1	-34.163	-5.820	13.794	-0.109760
ATOM	14	H7	C33	1	-34.015	-5.902	14.875	0.020476
ATOM	15	H8	C33	1	-35.194	-6.142	13.609	0.020476
ATOM	16	C3	C33	1	-33.196	-6.790	13.070	0.224041
ATOM	17	H5	C33	1	-32.912	-7.600	13.754	-0.061148
ATOM	18	H6	C33	1	-32.266	-6.258	12.840	-0.061148
ATOM	19	C2	C33	1	-33.748	-7.388	11.774	-0.003165
ATOM	20	H3	C33	1	-34.705	-7.879	11.991	-0.015740
ATOM	21	H4	C33	1	-33.989	-6.576	11.078	-0.015740
ATOM	22	C1	C33	1	-32.801	-8.395	11.099	-0.024887
ATOM	23	H1	C33	1	-31.766	-8.067	11.256	0.001481
ATOM	24	H2	C33	1	-32.941	-8.359	10.014	0.001481
ATOM	25	C11	C33	1	-32.945	-9.849	11.597	0.111481
ATOM	26	H14	C33	1	-33.564	-9.870	12.502	-0.031141
ATOM	27	H15	C33	1	-33.498	-10.443	10.860	-0.031141

ATOM	28	C12	C33	1	-31.613	-10.547	11.887	-0.035803
ATOM	29	H16	C33	1	-31.060	-9.967	12.630	-0.004269
ATOM	30	H17	C33	1	-30.990	-10.508	10.986	-0.004269
ATOM	31	C13	C33	1	-31.752	-12.019	12.349	0.174430
ATOM	32	H18	C33	1	-32.817	-12.292	12.380	-0.023858
ATOM	33	H19	C33	1	-31.332	-12.666	11.573	-0.023858
ATOM	34	C14	C33	1	-31.125	-12.414	13.670	-0.205150
ATOM	35	H20	C33	1	-30.545	-13.334	13.632	0.094084
ATOM	36	C15	C33	1	-31.233	-11.838	14.876	-0.219104
ATOM	37	H21	C33	1	-30.700	-12.310	15.698	0.089388
ATOM	38	C16	C33	1	-32.084	-10.661	15.289	0.419071
ATOM	39	H22	C33	1	-32.982	-11.060	15.781	-0.060562
ATOM	40	H23	C33	1	-32.464	-10.127	14.415	-0.060562
ATOM	41	C17	C33	1	-31.424	-9.694	16.253	-0.309863
ATOM	42	H24	C33	1	-31.985	-9.477	17.159	0.113659
ATOM	43	C18	C33	1	-30.242	-9.085	16.107	-0.168055
ATOM	44	H25	C33	1	-29.909	-8.428	16.907	0.074942
ATOM	45	C19	C33	1	-29.305	-9.191	14.921	0.394374
ATOM	46	H26	C33	1	-29.260	-10.232	14.580	-0.056401
ATOM	47	H27	C33	1	-29.735	-8.637	14.078	-0.056401
ATOM	48	C20	C33	1	-27.908	-8.678	15.195	-0.236009
ATOM	49	H28	C33	1	-27.648	-7.713	14.766	0.111129
ATOM	50	C21	C33	1	-27.001	-9.317	15.933	-0.267977
ATOM	51	H29	C33	1	-27.209	-10.284	16.382	0.117665
ATOM	52	H30	C33	1	-26.017	-8.899	16.115	0.117665

Charge file for AMBER (.prepin)

1	DUMM	DU	M	0	-1	-2	0.000	.0	.0	.00000
2	DUMM	DU	M	1	0	-1	1.449	.0	.0	.00000
3	DUMM	DU	M	2	1	0	1.523	111.21	.0	.00000
4	O1	oh	M	3	2	1	1.540	111.208	-180.000	-0.566139
5	H13	ho	E	4	3	2	0.966	118.102	-55.713	0.406955
6	C9	ca	M	4	3	2	1.368	49.587	35.979	0.395426
7	C10	ca	S	6	4	3	1.398	122.415	-110.033	-0.364152
8	H12	ha	E	7	6	4	1.088	119.573	-0.030	0.119379
9	C8	ca	M	6	4	3	1.399	117.358	69.621	-0.245240
10	H11	ha	E	9	6	4	1.084	119.185	0.114	0.141465
11	C7	ca	M	9	6	4	1.392	119.048	-179.473	-0.040606
12	H10	ha	E	11	9	6	1.086	119.325	179.609	0.088642
13	C6	ca	M	11	9	6	1.396	120.921	-0.132	-0.238173
14	H9	ha	E	13	11	9	1.085	120.021	179.359	0.115187
15	C5	ca	M	13	11	9	1.401	120.311	-0.184	0.187428
16	C4	c3	M	15	13	11	1.513	121.082	-177.715	-0.109760
17	H7	hc	E	16	15	13	1.094	109.443	-146.997	0.020476

18	H8	hc	E	16	15	13	1.096	108.953	-31.768	0.020476
19	C3	c3	M	16	15	13	1.549	114.109	90.199	0.224041
20	H5	hc	E	19	16	15	1.098	109.411	146.479	-0.061148
21	H6	hc	E	19	16	15	1.096	108.896	31.478	-0.061148
22	C2	c3	M	19	16	15	1.530	114.537	-90.246	-0.003165
23	H3	hc	E	22	19	16	1.097	108.782	-55.296	-0.015740
24	H4	hc	E	22	19	16	1.096	109.118	59.186	-0.015740
25	C1	c3	M	22	19	16	1.538	113.912	-177.502	-0.024887
26	H1	hc	E	25	22	19	1.097	108.801	-34.989	0.001481
27	H2	hc	E	25	22	19	1.095	109.553	-148.829	0.001481
28	C11	c3	M	25	22	19	1.544	114.683	87.187	0.111481
29	H14	hc	E	28	25	22	1.097	109.690	-11.694	-0.031141
30	H15	hc	E	28	25	22	1.096	109.910	103.327	-0.031141
31	C12	c3	M	28	25	22	1.532	114.157	-134.371	-0.035803
32	H16	hc	E	31	28	25	1.093	109.084	57.635	-0.004269
33	H17	hc	E	31	28	25	1.096	108.812	-55.942	-0.004269
34	C13	c3	M	31	28	25	1.549	114.300	-178.374	0.174430
35	H18	hc	E	34	31	28	1.100	109.339	0.103	-0.023858
36	H19	hc	E	34	31	28	1.094	108.418	113.084	-0.023858
37	C14	c2	M	34	31	28	1.515	118.085	-124.056	-0.205150
38	H20	ha	E	37	34	31	1.088	114.245	-133.310	0.094084
39	C15	c2	M	37	34	31	1.341	129.722	50.286	-0.219104
40	H21	ha	E	39	37	34	1.087	116.997	178.994	0.089388
41	C16	c3	M	39	37	34	1.510	128.773	3.945	0.419071
42	H22	hc	E	41	39	37	1.099	107.456	99.650	-0.060562
43	H23	hc	E	41	39	37	1.092	110.992	-14.112	-0.060562
44	C17	c2	M	41	39	37	1.517	115.189	-140.458	-0.309863
45	H24	ha	E	44	41	39	1.087	115.613	-126.918	0.113659
46	C18	c2	M	44	41	39	1.338	127.262	53.274	-0.168055
47	H25	ha	E	46	44	41	1.087	117.733	-179.136	0.074942
48	C19	c3	M	46	44	41	1.515	126.872	1.918	0.394374
49	H26	hc	E	48	46	44	1.096	109.589	-41.641	-0.056401
50	H27	hc	E	48	46	44	1.097	108.899	72.552	-0.056401
51	C20	c2	M	48	46	44	1.513	113.920	-165.215	-0.236009
52	H28	ha	E	51	48	46	1.088	116.748	-105.055	0.111129
53	C21	c2	M	51	48	46	1.333	124.479	73.932	-0.267977
54	H29	ha	E	53	51	48	1.086	121.701	1.184	0.117665
55	H30	ha	E	53	51	48	1.084	121.716	-178.901	0.117665

Table S1. Energy decomposition by the MM/GBSA method for cardanol compounds, highlighting the main residues contributing to binding within the catalytic active site (CAS) and the peripheral anionic site (PAS) of the enzyme.

Residue	**C1**	**C2**	**C3**
PAS			
Y72	-0.07 ± 0.01	-0.42 ± 0.32	-0.11 ± 0.01
Y124	-1.02 ± 0.04	-1.60 ± 0.30	-1.25 ± 0.05
W286	-0.54 ± 0.06	-0.46 ± 0.25	-0.56 ± 0.12
F295	-0.66 ± 0.36	-1.65 ± 0.50	-1.06 ± 0.05
Y341	-2.27 ± 0.09	-2.30 ± 0.38	-2.50 ± 0.44
CAS			
W86	-1.26 ± 0.40	-0.22 ± 0.18	-1.95 ± 0.38
S203	-0.61 ± 0.04	-0.59 ± 0.04	-0.98 ± 0.04
F297	-0.81 ± 0.28	-1.65 ± 0.27	-1.42 ± 0.29
F338	-2.08 ± 0.06	-2.07 ± 0.05	-2.29 ± 0.62
H447	-1.01 ± 0.34	-0.66 ± 0.36	-1.66 ± 0.18

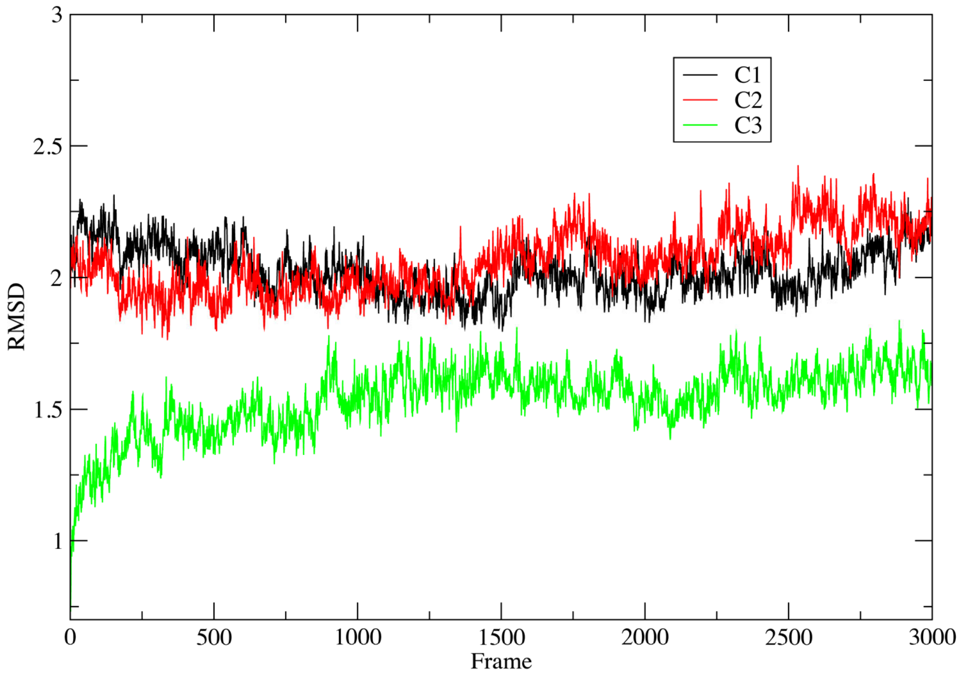

Figure S1. Root mean square deviation (RMSD) of complexes **C1**, **C2**, and **C3** over 100 ns of molecular dynamics simulations. The RMSD profiles indicate the structural stability and equilibration behavior of each system throughout the simulation time.
